# Antimicrobial Properties of Some Bis(Iminoacenaphthene (BIAN)-Supported *N*-Heterocyclic Carbene Complexes of Silver and Gold

**DOI:** 10.3390/molecules16032285

**Published:** 2011-03-09

**Authors:** Rachel R. Butorac, Salem S. Al-Deyab, Alan H. Cowley

**Affiliations:** 1 Department of Chemistry and Biochemistry, University of Texas at Austin, University Station, A5300, Austin, TX 78712, USA; 2 Chemistry Department, College of Science, King Saud University, P.O. Box 2455, Riyadh 11451, Saudi Arabia

**Keywords:** *N*-heterocyclic carbene, gold, silver, antimicrobial

## Abstract

The AgCl, AgOAc, AuCl, and AuOAc complexes of the new bis(imino)acenaphthene(BIAN)-supported *N*-heterocyclic carbene ligand and the precursor imidazolium salt have been investigated with respect to their antimicrobial activities against *Staphylococcus aureus*, *Bacillus subtilis*, *Escherichia coli* and *Psudomonas aeruginosa*. The most active antimicrobial is the precursor imidazolium salt, which has a minimum inhibitory concentration (MIC) value of <40 μg/mL. The MIC values for the silver complexes IPr(BIAN)AgCl and IPr(BIAN)AgOAc against Gram-positive *S. aureus* are comparable to that for AgNO_3_, while those against Gram-negative *E. coli* and *P. aeroginosa* are significantly larger. Similar behavior was evident for the gold acetate complex IPr(BIAN)AuOAc. However, in the case of the gold chloride analogue, the MIC values are virtually identical for both the Gram-positive and the Gram-negative bacteria.

## 1. Introduction

Recently, we reported the synthesis and structure of the first example of an *N*-heterocyclic carbene supported by a bis(imino)acenaphthene (BIAN) ligand [1], along with a number of Ag, Au and Ir coordination compounds. Given the usefulness of NHC complexes of these and other metals as anti-microbials [2], we became interested in exploring the properties of some of the corresponding BIAN compounds. The BIAN class of ligand, which features the fusion of a naphthalene moiety to a diimine, has a number of interesting and distinctive properties [3]. One such property is its extensive redox behavior. For example, BIAN ligands are capable of sequentially accommodating up to four electrons. These redox properties of the BIAN ligand class could, in principle, be useful for controlling the metal release from e.g. antimicrobials and anti-cancer drugs. An additional desirable property common to both NHC and BIAN carbene ligands is that the nitrogen substituents can be varied over a wide range. Lipophilicity can also play an important role in antimicrobial activity. For example, it is known that the activity of NHC imidazolium salts is enhanced significantly if long chain alkyl or bulky aryl groups are employed as *N*-substituents [4]. In the present paper, we explore the antimicrobial properties of the precursor IPr-BIAN imidazolium salt (**1**), along with those of the AgCl (**2**), AgOAc (**3**), AuCl (**4**) and AuOAc (**5**) complexes of the new BIAN carbene ligand. 

## 2. Results and Discussion

### 2.1. Syntheses of 1-5 and the X-ray Crystal Structure of 5

**Scheme 1:**
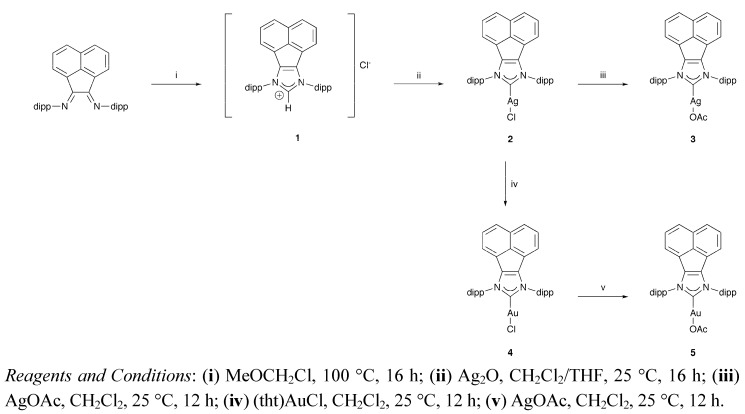
Synthetic pathways to **1**-**5**.

Compounds **1**, **2** and **4** were prepared as described previously [1]. Compounds **3** and **5** are new and were prepared by treatment of **2** and **4**, with AgOAc and AuOAc, respectively, in CH_2_Cl_2_ solution (Scheme 1). Both compounds were characterized on the basis of MS, HRMS and multinuclear NMR data.

**Figure 1:**
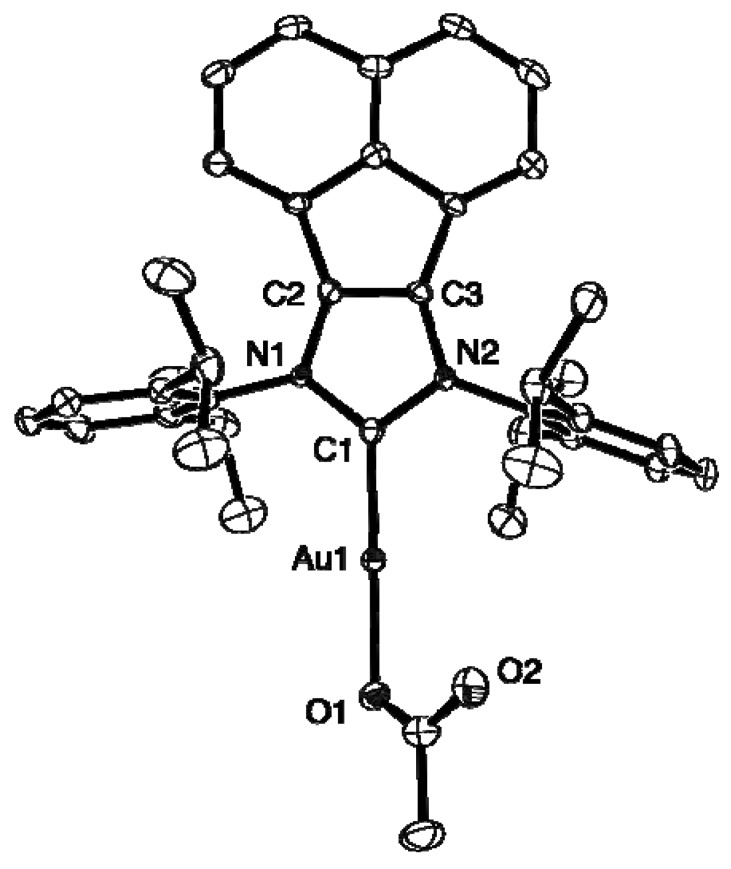
ORTEP diagram of **5** with 30% probability thermal ellipsoids. Hydrogen atoms are omitted for clarity.

The gold complex **5** was also characterized by single-crystal X-ray diffraction. A summary of X-ray data collection details appears in Table 1 and a selection of pertinent metrical parameters is presented in Table 2. The C(2)-C(3) bond distance for the NHC moiety of **5** (1.352(6) Å) is virtually identical to that of the AuCl analogue **4** (1.358(4) Å) and indicative of some double bond character, as are the average C-N bond distances of the NCCN fragment (Figure 1).

**Table 1:** Selected crystal data, data collection and refinement parameters for compound **5**.

Formula	C_40_H_45_AuCl_2_N_2_O_2_
Formula weight	853.65
Crystal system	Monoclinic
Space group	P2_1_/c
*a*/Å	12.764(2)
*b*/Å	19.274(3)
*c*/Å	15.025(3)
*α*/°	90.0
*β*/°	93.849(5)
*γ*/°	90.0
Z	4
*D_c_*/g cm^-3^	1.537
F(000)	1712
Crystal size/nm	0.27x0.11x0.10
*Θ* range/°	3.03-26.00
Collected reflections	7234
Independent reflections	7234
*R*_1_ [*I*>2σ(*I*)]	0.0318
w*R*_2_ (all data)	0.0796

**Table 2:** Selected bond distances (Å) and angles (°) for compound **5**.

Bond distances/Å		Bond angles/°	
C(1)-N(1)	1.357(5)	N(1)-C(1)-N(2)	105.6(3)
C(1)-N(2)	1.365(5)	N(1)-C(2)-C(3)	107.8(4)
C(2)-C(3)	1.352(6)	C(2)-C(3)-N(2)	106.9(3)
C(2)-N(1)	1.360(5)	C(1)-N(1)-C(2)	110.1(3)
C(3)-N(2)	1.377(5)	C(1)-N(2)-C(3)	109.6(3)
C(1)-Au(1)	1.949(4)	N(1)-C(1)-Au(1)	129.5(3)
Au(1)-O(1)	2.023(3)	N(2)-C(1)-Au(1)	124.9(3)
		C(1)-Au(1)-O(1)	177.62(14)

### 2.2. Antimicrobial Testing of Compounds ***1-5***

Compounds **1**-**5** were tested for their antimicrobial activities against the Gram-positive bacterial strains *Staphylococcus aureus* and* Bacillus subtilis* and the Gram-negative bacterial strains *Escherichia coli* and *Pseudomonas aeruginosa* by determination of their corresponding minimum inhibitory concentration (MIC) values [5] in Mueller-Hinton (MH) broth. Compounds **1**-**5** were dissolved initially in a 4% dimethyl sulfoxide (DMSO)-water solution, followed by serial dilutions with the MH broth while adding additional DMSO in order to maintain a 2% DMSO concentration in each dilution.

The results of the MIC tests for **1**-**5** are summarized in Table 3. Clearly, the most active antimicrobial for both the Gram-positive and Gram-negative bacteria is the BIAN imidazolium salt **1 **with a MIC value of < 40 μg/mL for each pathogen. Interestingly, and despite the efficacy of imidazolium salts, relatively little has been published in this area. Early work by Pernak *et al*. [4] established that the MIC values for *N*-alkylthiomethyl-substituted imidazolium salts are dependent upon the length of the hydrocarbon chain attached to the sulfur atom. The optimum chain lengths were found to be C_10_H_21_ and C_12_H_25_ and the use of longer or shorter chains resulted in diminished antibacterial activity. The MIC ranges were found to be 1.13−11.9 and 0.69−46 μg/mL for the C_10_H_21_ and C_12_H_25_ derivatives, respectively. Somewhat similar results have been reported more recently by Lee et al. [6]. In a subsequent publication, Pernak *et al*. [7] reported that the MIC values for the 3-alkoxymethyl analogues were not particularly sensitive to the type of counter-anion. Working with saturated 1,3-diazolidinium salts, Çetkinkaya *et al*. [8] discovered that, while there was a dependence of activity on the length of the *n*-alkyl chains, the attachment of mesityl or mesitylmethyl substituents to the N atoms resulted in the most significant increase of activity towards both Gram-positive and Gram-negative bacteria. Interestingly, however, in contrast to the work of Pernak *et al*. [4] the activity of these derivatives was found to depend on the identity of the counter anion. For example, the value for the mesitylmethyl chloride salt was 3.12 μg/mL.

**Table 3:** Minimum inhibitory concentrations (MIC) for compounds **1**-**5 **in μg/mL.

Compound	*S. aureus*	*B. subtilis*	*E. coli*	*P. aeruginosa*
Imidazolium Salt (**1**)	< 40	< 40	< 40	< 40
IPr(BIAN)AgCl (**2**)	310	310	> 20,000	5,000
IPr(BIAN)AgOAc (**3**)	630	1,300	10,000	10,000
IPr(BIAN)AuCl (**4**)	630	630	630	630
IPr(BIAN)AuOAc (**5**)	630	630	> 20,000	10,000

The first examples of Ag (I)-substituted NHC complexes featuring antimicrobial activity against some common pathogens were reported by Youngs *et al*. [2] and involve a one-dimensional polymeric arrangement of pyridine-supported pincer ligands featuring pendant (NHC)Ag^+^ moieties. These polymers were tested against *E. coli*, *S. aureus* and *P. aeruginosa* and in each case the activity against the pathogen exceeded that of AgNO_3_ (for AgNO_3_, a MIC value of 433 μg/mL was cited). Comparison with the present results (Table 3) reveals that while the MIC values for IPr(BIAN)AgCl and IPr(BIAN)AgOAc [9] against Gram-positive *S. aureus* are comparable to that for AgNO_3 _, those for Gram-negative *E. coli* and *P. aeroginosa* are larger.

Analogously to the present work, the AgCl complex of 1-benzyl-3-*tert*-butylimidazol-2-ylidene reported by Ghosh *et al*. [10] was found to be active against Gram-positive *B. subtilis*, (MIC = 25 ± 3.2 μM). However, there was no evidence of antimicrobial activity against Gram-negative *E. coli*. Interestingly however, the precursor imidazolium salt was found to be inactive. The corresponding AuCl complex was found to be more active against *B. subtilis* (MIC = 15 ± 2.3 μM) than the AgCl analogue. The reason cited for the superior antimicrobial activity of the gold complex is based on the fact that DFT calculations revealed that the NHC-Au bond strength is superior to that of the Ag analogue. Relatively little has been published on the antimicrobial behavior of other NHC gold (I) complexes [11]. In 2004, Çetinkaya *et al.* [12] synthesized and explored the antimicrobial behavior of gold salts of the general type [Au(NHC)_2_][Cl] in which the NHCs were imidazolidine-2-ylidenes bearing alkyl or aryl-*N*-substituents. A marked dependence was noted on the nature of the nitrogen substituents in the sense that changing the *para* substituent of the *N*-benzyl groups resulted in substantial differences in activity with respect to both Gram-positive and Gram-negative bacteria. Thus, while the *para*-trimethoxyphenyl and *para*-*tert*-butylphenyl derivatives had MIC values of 12.5 μg/mL toward *S. aureus*, that of the *para*-pentamethylphenyl analogue was 200 μg/mL, which is comparable to those found in the present work for **4** and **5** (630 μg/mL). In the most recent work, three substituted benzimidazole gold(I) chloride complexes were synthesized and tested against a selection of Gram-positive and Gram-negative bacteria and fungi. A marked dependence of antimicrobial activity on the nature of the *N*-aryl substituents was evident. Thus, while the bis(trimethoxybenzyl) and the trimethoxybenzyl/*para*-*tert*-butyl derivatives exhibited MIC values of 12.5 μg/mL, that of the bis(pentamethyl)benzyl analogue was 200 μg/mL [13] which is comparable with the 630 μg/mL MIC values found for **4** and **5** in the present work. Based on the foregoing, it is clear that the stereoelectronics of the NHC ligands can also play a very important role in terms of the antimicrobial activity.

## 3. Experimental

### 3.1. General

All glassware was oven-dried before use. All reagents were obtained commercially and used without further purification. Toluene and hexanes were dried over Na and freshly distilled prior to use. The dichloromethane was dried over CaH_2_ and freshly distilled prior to use. Compounds **1**, **2** and **4** were synthesized according to literature procedures [1].

### 3.2. Physical Measurements

Low-resolution CI mass spectra were obtained on a Thermo Scientific TSQ Quantum GC mass spectrometer and high-resolution CI mass spectra were recorded on a magnetic sector Waters Autospec Ultima instrument. ^1^H- and ^13^C{^1^H}-NMR spectra were recorded at 295 K in CDCl_3_ on a Varian Unity 300 (^1^H, 300 MHz; ^13^C, 75 MHz) or a Varian INOVA 500 spectrometer (^1^H, 500 MHz; ^13^C, 125 MHz) immediately following sample preparation. Deuterated chloroform was obtained from Cambridge Isotopes and stored over 4 Å molecular sieves prior to use. ^1^H and ^13^C{^1^H} chemical shift values are reported in parts per million (ppm) relative to SiMe_4_ (δ 0.00), using the solvent resonance as the internal standard.

### 3.3. X-Ray Crystallography

For compound **5**, a crystal of suitable quality was removed from a vial, covered with mineral oil and mounted on a nylon thread loop. The X-ray diffraction data were collected on a Rigaku AFC-12 Saturn 724+ CCD diffractometer equipped with a graphite-monochromated Mo Kα radiation source (λ = 0.71073 Å) and a Rigaku XStream low temperature device cooled to 100 K. Corrections were applied for Lorentz and polarization effects. The structure was solved by direct methods and refined by full-matrix least-squares cycles on F^2^ using the Siemens SHELXTL PLUS 5.0 (PC) software package [14]. All non-hydrogen atoms were refined anisotropically, and hydrogen atoms were placed in fixed, calculated positions using a riding model. The CCDC reference number for compound **5** is 793378.

### 3.4. Preparations

*IPr(BIAN)[AgOAc]* (**3**). Dichloromethane (30 mL) was added to a 50 mL round bottom flask that had been covered with aluminum foil, then charged with **2** (0.115 g, 0.175 mmol) and AgOAc (0.030 g, 0.179 mmol). The reaction mixture was stirred for 12 h at ambient temperature, after which the solvent was removed under reduced pressure. The crude product was digested in toluene, filtered and the solvent stripped under reduced pressure to afford analytically pure **3** as a bright yellow solid (0.0775 g, 65.1%). MS (CI^+^, CH_4_): *m/z* 679 [M+H]^+^; HRMS (CI^+^, CH_4_): calcd for C_39_H_44_N_2_O _2_Ag *m/z* 679.2454; found, 679.2449; ^1^H-NMR: *δ* 1.11 (d, 12H, CH_3_), 1.33 (d, 12H, CH_3_), 1.88 (s, 3H, OAc-CH_3_), 2.83 (sept, 4H, -CH), 7.02 (d, 2H, Naph-H), 7.40 (d, 4H, Ar-H), 7.43 (t, 2H, Naph-H), 7.59 (t, 2H, Ar-H), 7.81 (d, 2H, Naph-H); ^13^C-NMR: *δ* 22.66, 23.83, 24.69, 28.88, 121.05, 124.57, 125.27, 127.79, 128.37, 129.86, 130.70, 130.84, 133.37, 139.19, 139.25, 145.63, 178.07; mp (decomp): 268-270 °C.

*IPr(BIAN)[AuOAc]* (**5**). Dichloromethane (30 mL) was added to a 50 mL aluminum foil-wrapped round bottom flask containing **4** (0.120 g, 0.161 mmol) and AgOAc (0.0280 g, 0.168 mmol). The reaction mixture was stirred for 12 h at ambient temperature, after which the solvent was removed under reduced pressure. The crude product was digested in toluene, filtered and the solvent stripped under reduced pressure to afford analytically pure **5** as a bright yellow solid (0.0735 g, 59.3%). A suitable crystal for X-ray diffraction analysis was grown by slow evaporation of a saturated DCM/hexanes solution of **5**. MS (CI^+^, CH_4_): *m/z* 769 [M+H]^+^; HRMS (CI^+^, CH_4_): calcd for C_39_H_44_N_2_O_2_Au *m/z* 769.3068; found, 769.3068; ^1^H-NMR: *δ* 1.10 (d, 12H, CH_3_), 1.41 (d, 12H, CH_3_), 1.81 (s, 3H, OAc-CH_3_), 2.82 (sept, 4H, -CH), 7.01 (d, 2H, Naph-H), 7.40 (d, 4H, Ar-H), 7.44 (t, 2H, Naph-H), 7.60 (t, 2H, Ar-H), 7.81 (d of d, 2H, Naph-H);^ 13^C-NMR: *δ* 23.91, 23.96, 24.38, 29.05, 121.21, 124.52, 125.36, 127.80, 128.55, 129.78, 130.29, 130.87, 132.83, 138.05, 145.67, 176.35; mp (decomp): 340-343 °C.

### 3.5. Antimicrobial Activities of ***1-5***

Antimicrobial activities of compounds **1**-**5** were determined using the Microtiter-Based Minimum Inhibitory Concentration (MIC) Test. *S. aureus* ATCC 6538, *B. subtilis* ATCC 19659, *E. coli* ATCC 11229 and *P. aeruginosa* ATCC 15442 were grown to approximetly 10^5^ CFU/mL in Mueller-Hinton broth. The stock solutions of compounds **1**-**5** were prepared with DI water supplemented with dimethyl sulfoxide (DMSO). The concentrations of the tested compounds ranged from 2% to 0.0039% using the Mueller-Hinton broth as the diluent, supplemented with DMSO to a final concentration of 2%. The plates were incubated at 36.0 ± 1 °C for 18-24 h. The MIC was taken to be the last well in the dilution series that did not exhibit growth, determined on the basis of turbidity.

## 4. Conclusions

The first examples of silver and gold antimicrobial compounds supported by the BIAN ligand class are reported. The antimicrobial activities of these complexes and the precursor imidazolium salt have been investigated. In terms of Minimum Inhibitory Concentration (MIC) values, the most active compound against both Gram-positive and Gram-negative bacteria is the precursor imidazolium salt. Comparison of the MIC values for the AgCl and AgOAc ligated complexes reveals that their activities against Gram-positive *S. aureus* are similar to that of AgNO_3_. On the other hand, these complexes are significantly less active toward Gram-negative *E. coli* and *P. aeroginosa*.
